# Comparative study of stewardship of road traffic injuries prevention with a focus on the role of ‎health system: Three pioneer countries and three similar to Iran

**Published:** 2019-08

**Authors:** Naser Derakhshani, Ramin Rezapour, Ghader Darghahi, Mehdi Nikoomanesh, Hojat Gharaee

**Affiliations:** ^*a*^Student Research Committee, Iran University of Medical Sciences, Tehran, Iran.; ^*b*^Road Traffic Injury Research Center, Tabriz University of Medical Sciences, Tabriz, Iran.; ^*c*^Tabriz Health Services Management Research Center, Health Management and Safety Promotion Research Institute, Tabriz University of Medical Sciences, Tabriz, Iran.

## Abstract

**Background:**

Road traffic injuries (RTIs) are one of the main public health problems in almost all countries ‎which lead to 1.2 million ‎deaths and 50 million injuries annually. In this regard, studying the ‎role of involved organizations and the health ‎system can be helpful. So this study is performed ‎with the aim of comparing the stewardship of RTIs prevention in ‎three pioneer countries and ‎three similar ones to Iran. ‎ ‎

**Methods:**

In this descriptive comparative study, the United States of America, Sweden, and Brazil as the ‎pioneer countries in RTIs prevention were compared to the India, Pakistan, and Turkey as the ‎countries socioeconomically similar to Iran. Embase, PubMed, Scopus, IranDoc, IranMedex, ‎SID, and MagIran were searched. Also a hand search conducted on websites and search engines ‎using related keywords. ‎

**Results:**

In the pioneer countries in RTIs prevention there was a delegation to a particular organization. ‎In the other three countries a part of the Ministry of Transportation had the overall ‎responsibility of RTIs. In Iran there was uncertainty in the stewardship of RTIs prevention. ‎There was little evidence on the role and activities of health systems in RTIs prevention.‎

**Conclusions:**

It seems necessary to define a lead agency organization on RTIs prevention in Iran with ‎sufficient authority and resources. This study also recommends conducting reliable studies to ‎investigate the possible roles that the health system of a country can assume regarding the RTIs ‎prevention.‎

**Keywords:**

Road traffic injuries, RTIs, Comparative study, ‎

